# Crystal structure of *catena*-poly[[[di­aqua­bis­(2,4,6-tri­methyl­benzoato-κ*O*)cobalt(II)]-μ-aqua-κ^2^ *O*:*O*] dihydrate]

**DOI:** 10.1107/S2056989017005564

**Published:** 2017-04-13

**Authors:** Tuncer Hökelek, Nurcan Akduran, Safiye Özkaya, Hacali Necefoğlu

**Affiliations:** a Department of Physics, Hacettepe University, 06800 Beytepe, Ankara, Turkey; b SANAEM, Saray Mahallesi, Atom Caddesi, No. 27, 06980 Saray-Kazan, Ankara, Turkey; c Department of Chemistry, Kafkas University, 36100 Kars, Turkey; d International Scientific Research Centre, Baku State University, 1148 Baku, Azerbaijan

**Keywords:** crystal structure, cobalt(II), transition metal complex, benzoic acid derivative

## Abstract

The asymmetric unit of the title one-dimensional polymeric compound, {[Co(C_10_H_11_O_2_)_2_(H_2_O)_3_]·2H_2_O}_*n*_, contains one Co^II^ cation situated on a centre of inversion, one-half of a coordinating water mol­ecule, one 2,4,6-tri­methyl­benzoate (TMB) anion together with one coordinating and one non-coordinating water mol­ecule; the TMB anion acts as a monodentate ligand. In the anion, the carboxyl­ate group is twisted away from the attached benzene ring by 84.9 (2)°. The Co^II^ atom is coordinated by two TMB anions and two water mol­ecules in the basal plane, while another water mol­ecule bridges the Co^II^ atoms in the axial directions, forming polymeric chains running along [001]. The coordination environment for the Co^II^ cation is a slightly distorted octa­hedron. The coordinating and bridging water mol­ecules link to the carboxyl­ate groups *via* intra- and inter­molecular O—H⋯O hydrogen bonds, enclosing *S*(6) ring motifs, while the coordinating, bridging and non-coordinating water mol­ecules link to the carboxyl­ate groups and the coordinating water mol­ecules link to the non-coordinating water mol­ecules *via* O—H⋯O hydrogen bonds, enclosing *R*_2_^2^(8) and *R*_3_^3^(8) ring motifs. Weak C—H⋯O and C—H⋯π inter­actions may further stabilize the crystal structure.

## Abstract 2

In {[Co(C_10_H_11_O_2_)_2_(H_2_O)_3_]·2H_2_O}_*n*_, the Co^II^ atom is coordinated by two TMB anions and two water mol­ecules in the basal plane, while another water mol­ecule bridges the Co^II^ atoms in the apical directions, forming polymeric chains running along [001].

## Chemical context   

Transition metal complexes with ligands of biochemical inter­est, such as imidazole and some N-protected amino acids, show inter­esting physical and/or chemical properties, through which they may find applications in biological systems (Antolini *et al.*, 1982 ▸). Some benzoic acid derivatives, such as 4-amino­benzoic acid, have been extensively reported in coordination chemistry, as bifunctional organic ligands, due to the varieties of their coordination modes (Chen & Chen, 2002 ▸; Amiraslanov *et al.*, 1979 ▸; Hauptmann *et al.*, 2000 ▸).

The structure–function–coordination relationships of the aryl­carboxyl­ate ion in Zn^II^ complexes of benzoic acid deriv­atives change depending on the nature and position of the substituted groups on the benzene ring, the nature of the additional ligand mol­ecule or solvent, and the pH and temperature of the synthesis (Shnulin *et al.*, 1981 ▸; Nadzhafov *et al.*, 1981 ▸; Antsyshkina *et al.*, 1980 ▸; Adiwidjaja *et al.*, 1978 ▸). When pyridine and its derivatives are used instead of water mol­ecules, the structure is completely different (Catterick *et al.*, 1974 ▸).

The solid-state structures of anhydrous zinc(II) carboxyl­ates include one-dimensional (Guseinov *et al.*, 1984 ▸; Clegg *et al.*, 1986*a* ▸), two-dimensional (Clegg *et al.*, 1986*b* ▸, 1987 ▸) and three-dimensional (Capilla & Aranda, 1979 ▸) polymeric motifs of different types, while discrete monomeric complexes with octa­hedral or tetra­hedral coordination geometry are found if water or other donor mol­ecules coordinate to the Zn^II^ cation (van Niekerk *et al.*, 1953 ▸; Usubaliev *et al.*, 1992 ▸).
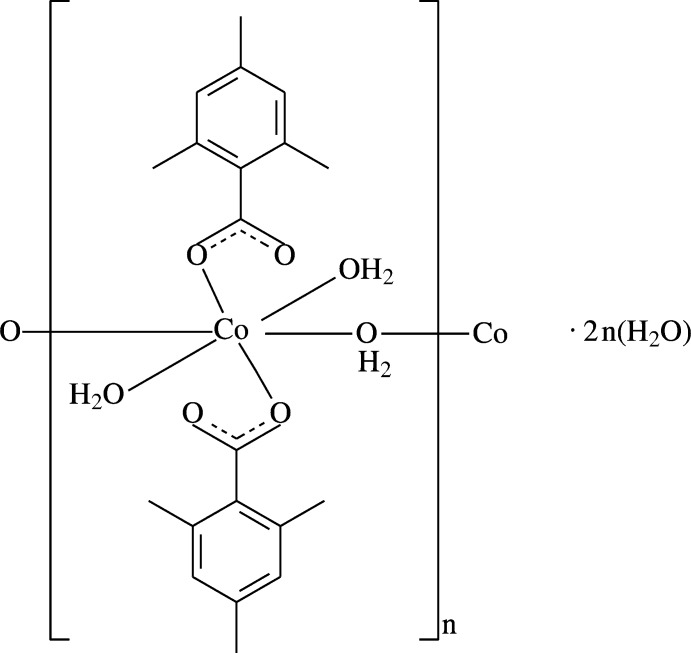


The structures of some mononuclear polymeric complexes obtained from the reactions of transition metal(II) ions with nicotinamide (NA) and/or some benzoic acid derivatives as ligands have been determined, *e.g.* {Mn(C_11_H_14_NO_2_)_2_(H_2_O)_3_·2H_2_O}_*n*_ [(II); Hökelek *et al.*, 2009 ▸)], [Mn(C_7_H_4_FO_2_)_2_(H_2_O)]_*n*_ [(III); Necefoğlu *et al.*, 2011 ▸)], {[Pb(C_9_H_9_O_2_)_2_(C_6_H_6_N_2_O)]·H_2_O}_*n*_ [(IV); Hökelek *et al.*, 2011 ▸)], {[Pb(C_7_H_5_O_3_)_2_(C_6_H_6_N_2_O)]H_2_O}_*n*_ [(V); Zaman *et al.*, 2012 ▸)] and {[Zn(C_7_H_4_ClO_2_)_2_(H_2_O)]}_*n*_ [(VI); Bozkurt *et al.*, 2013 ▸)], where the transition metal(II) cations are bridged by water mol­ecules in (II), 4-fluoro­benzoate anions in (III), nicotinamide ligands in (IV), 3-hy­droxy­benzoate anions in (V) and 3-chloro­benzoate anions in (VI). The synthesis and structure determination of the title compound, (I), a one-dimensional polymeric cobalt(II) complex with two 2,4,6-tri­methyl­benzoate (TMB) ligands and four coordinating and two non-coordinating water mol­ecules, was undertaken in order to compare the results obtained with those reported previously. Its crystal structure is reported herein.

## Structural commentary   

**Figure 1:**
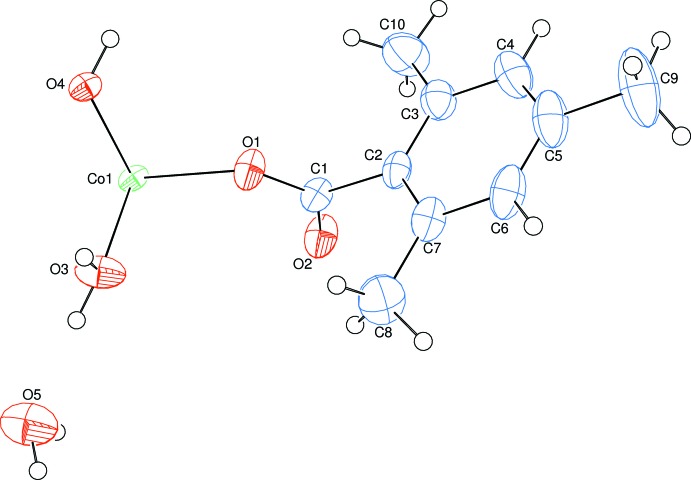
The asymmetric unit of the title mol­ecule with the atom-numbering scheme. Displacement ellipsoids are drawn at the 50% probability level.

The asymmetric unit of the title one-dimensional polymeric compound, (I), contains one Co^II^ cation situated on a centre of inversion, one-half of a coordinating water mol­ecule, one 2,4,6-tri­methyl­benzoate (TMB) anion together with the one coordinating and one non-coordinating water mol­ecules; the TMB anion acts as a monodentate ligand (Fig. 1 ▸).

**Figure 2:**
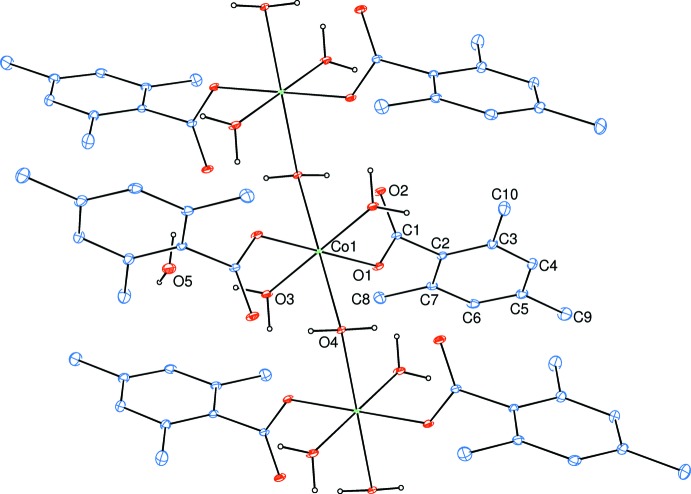
Partial view of the polymeric chain of the title compound. H atoms of the 2,4,6-tri­methyl­benzoate (TMB) anions have been omitted for clarity.

**Figure 3:**
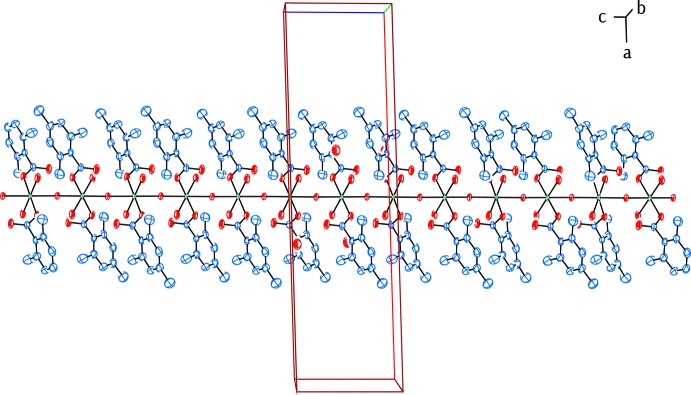
A partial packing diagram of the title one-dimensional polymeric compound in a view approximately along the *b* axis, where the *c* axis is horizontal and the *a* axis is vertical. H atoms have been omitted for clarity.

The Co^II^ atom is coordinated by two TMB anions and two water mol­ecules in the basal plane while another water mol­ecule bridges the Co^II^ atoms in the axial directions, resulting in a slightly distorted octa­hedral coordination sphere around each Co^2+^ cation, and forming polymeric chains (Fig. 2 ▸) running along [001] (Figs. 3 ▸ and 4 ▸). The cobalt cation is formally Co^2+^ within the structure, in line with the presence of bridging water molecules rather than bridging hydroxide groups. This is confirmed by softness-sensitive BVS calculations (Adams, 2001 ▸), which identify the BVS for the Co atom to be 2.05 (5).

The two carboxyl­ate O atoms (O1 and O1^i^) of the two symmetry-related TMB anions and the two symmetry-related water O atoms (O3 and O3^i^) around the Co^II^ cation form a slightly distorted square-planar arrangement with an average Co1—O bond length of 2.058 (2) Å. The slightly distorted octa­hedral coordination is completed by the symmetry-related bridging O atoms (O4 and O4^i^) with a Co1—O4 bond length of 2.2060 (11) Å in the axial directions (Fig. 2 ▸) [symmetry code: (i) 1 − *x*, 1 − *y*, 1 − *z*]. The Co—O bond lengths are in the range of 2.041 (2)–2.2060 (11) Å. Among the Co—O coordinations the Co1—O3 bond [2.041 (2) Å] is the shortest and the Co1—O4 bond [2.2060 (11) Å] is the longest, probably as a result of the bidentate bridging coordination of O4 with a very wide Co1—O4—Co1^ii^ bond angle of 132.95 (13)° [symmetry code: (ii) 1 − *x*, *y*, 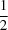 − *z*]. The Co1 atom lies 0.2077 (1) Å above the carboxyl­ate (O1/O2/C1) group, which makes a dihedral angle of 84.9 (2)° with the adjacent benzene (C2–C7) ring.

Neighboring Co^II^ atoms are bridged by H_2_O mol­ecules (Fig. 2 ▸) and they are also coordinated by monodentate carboxyl­ate groups. The non-coordinating oxygen atoms of the carboxyl­ate groups inter­act with the bridging water mol­ecules *via* short hydrogen bonds (Table 1 ▸ and Fig. 5 ▸), increasing the Lewis basicity of the bridging water mol­ecules by attracting the protons of the water mol­ecules to the oxygen atoms of the carboxyl­ate groups. Intra­molecular O—H_brdW_⋯O_c_ and inter­molecular O—H_coordW_⋯O_c_ (brdW = bridging water, coordW = coordinating water and c = carboxyl­ate) hydrogen bonds (Table 1 ▸) link the bridging and coordinating water mol­ecules to the carboxyl­ate oxygen atoms, enclosing *S*(6) ring motifs (Fig. 5 ▸).

## Supra­molecular features   

**Figure 4:**
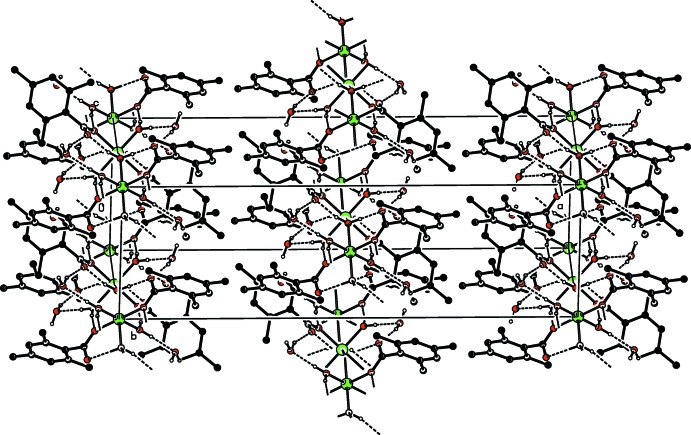
View of the hydrogen bonding and packing of the title one-dimensional polymeric compound along the *c* axis. H atoms not involved in classical hydrogen bonds have been omitted for clarity.

**Figure 5:**
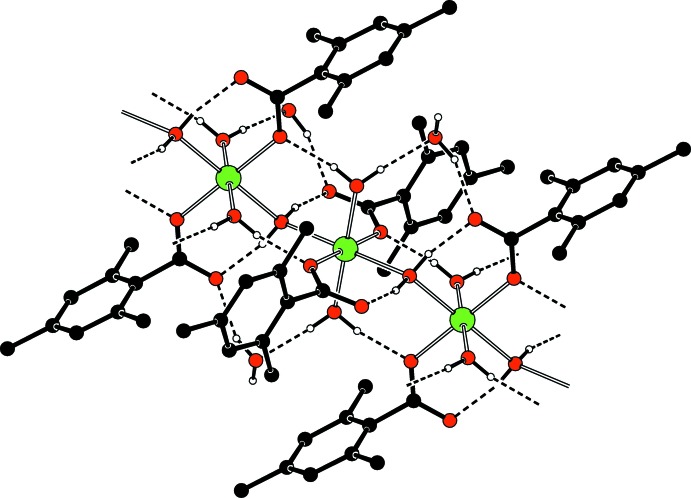
Part of the crystal structure. Intra­molecular and inter­molecular O—H⋯O hydrogen bonds, enclosing *S*(6), 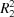
(8) and 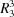
(8) ring motifs, are shown as dashed lines. H atoms not involved in classical hydrogen bonds have been omitted for clarity.

In the crystal, O—H_coordW_ ⋯ O_c_ and O—H_coordW_⋯O_noncoordW_, O—H_noncoordW_⋯O_c_, O—H_brdW_⋯O_c_ (noncoordW = non-coordinating water) hydrogen bonds (Table 1 ▸) link the mol­ecules, enclosing 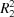
(8) and 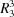
(8) ring motifs, respectively (Fig. 5 ▸). O—H⋯O hydrogen bonds (Table 1 ▸) also link the hydrogen-bonded polymeric chains running along [001] into networks parallel to (011) (Fig. 4 ▸). The crystal structure is further stabilized by weak C—H⋯O and C—H⋯π inter­actions (Table 1 ▸).

## Comparison with related structures   

In the crystal structure of a similar complex, *catena*-poly[[[di­aqua­bis­[4-(di­ethyl­amino)­benzoato-*κO^1^*]mang­anese(II)]-μ-aqua]­dihydrate], {[Mn(C_11_H_14_NO_2_)_2_(H_2_O)_3_]·2(H_2_O)}_*n*_, (II), (Hökelek *et al.*, 2009 ▸), the two independent Mn^II^ atoms are located on a centre of symmetry and are coordinated by two 4-(di­ethyl­amino)­benzoate (DEAB) anions and two water mol­ecules in the basal plane, while another water mol­ecule bridges the Mn atoms in the axial directions, forming polymeric chains as in the title compound, (I). In (II), the Mn—O bond lengths are in the range 2.1071 (14)–2.2725 (13) Å. The Mn—O bond lengths [2.2725 (13) and 2.2594 (13) Å] for the bridging water mol­ecule are the longest with an Mn—O—Mn bond angle of 128.35 (6)°.

In the crystal structure of *catena*-[bis­(μ_2_-aqua)­tetra­aqua­tetra­kis­(2,4,6-tri­methyl­benzoato-O)dinickel(II) tetra­hydrate, {[Ni(C_10_H_11_O_2_)_2_(H_2_O)_3_]·2H_2_O}_*n*_, [(VII; Indrani *et al.*, 2009 ▸)], the two independent Ni^II^ atoms are located on a centre of symmetry and are coordinated by two 2,4,6-tri­methyl­benzoate (TMB) anions and two water mol­ecules in the basal plane, while another water mol­ecule bridges the Ni atoms in the axial directions, forming polymeric chains as in the title compound, (I). In (VII), the Ni—O bond lengths are in the range 2.0337 (15)–2.1316 (13) Å. The Ni—O bond lengths [2.1316 (13) and 2.1299 (13) Å] for the bridging water mol­ecule are the longest with an Ni—O—Ni bond angle of 134.65 (7)°.

We also solved the crystal structure of *catena*-poly[[[di­aqua­bis­(2,4,6-tri­methyl­benzoato-*κO^1^*)manganese(II)], {[Mn(C_10_H_11_O_2_)_2_(H_2_O)_3_]·2H_2_O}_*n*_, (VIII), which had previously been reported by Chen *et al.* (2007 ▸). In (VIII), the Mn^II^ atom and the bridging water O atom are located on a centre of symmetry and the Mn^II^ atom is coordinated by two 2,4,6-tri­methyl­benzoate (TMB) anions and two water mol­ecules in the basal plane, while another water mol­ecule bridges the Mn^II^ cations in the axial directions, forming polymeric chains as in the title compound, (I). The Mn—O bond lengths are in the range 2.1409 (15)–2.2734 (7) Å. The Mn—O bond length [2.2734 (7) Å] for the bridging water mol­ecule is the longest with an Mn—O—Mn bond angle of 128.41 (8)°.

In the title compound, (I), the near equalities of the C1—O1 [1.259 (4) Å] and C1—O2 [1.246 (4) Å] bonds in the carboxyl­ate groups indicate delocalized bonding arrangements, rather than localized single and double bonds. The O2—C1—O1 bond angle [124.5 (3)°] is increased slightly compared to the free acid [122.2°] due to the coordination of oxygen atom (O1) to the metal atom. The O2—C1—O1 bond angle may be compared with the corresponding values of 121.96 (18) and 122.35 (18)° in (II), 124.0 (2)° in (III), 120.6 (6) and 121.3 (7)° in (IV), 121.7 (2) and 121.9 (3)° in (V), 123.47 (14)° in (VI), 124.29 (18) and 124.33 (18)° in (VII) and 124.02 (16)° in (VIII). The benzoate ions coordinate to the metal atoms in a monodentate fashion in (II), (III), (VI), (VII) and (VIII), and they are bidentate in (IV) and (V).

The Co1⋯Co1^ii^ distance [4.045 (15) Å] across the chain (Fig. 2 ▸) and the Co1—O4—Co1^ii^ bond angle [132.95 (13)°] in (I) may be compared with the corresponding values of 4.079 (4) Å and 128.35 (6)° in (II), 4.951 (3) Å in (III), 9.795 (4) Å in (IV), 7.363 (4) Å in (V), 4.3798 (3) Å in (VI), 3.932 Å and 134.65 (7)° in (VII) and 4.049 (15) Å and 128.41 (8)° in (VIII). According to these results, when the transition metal(II) atoms are bridged by the water mol­ecules the *M*—O_brdW_—*M* (*M* = transition metal and brdW = bridging water) bond angles increase, while the *M*—O_brdW_ bond lengths decrease with increasing atomic number, *Z*, of the transition metal(II) atoms and the *M*⋯*M* distances across the polymeric chains are almost the same, independent of the type of anion coordinating to the metal(II) atoms.

## Synthesis and crystallization   

The title compound was prepared by the reaction of CoSO_4_·7H_2_O (0.70 g, 2.5 mmol) with sodium 2,4,6-tri­methyl­benzoate (0.93 g, 5 mmol) in H_2_O (150 ml) at room temperature. The mixture was set aside to crystallize at ambient temperature for eight weeks, giving pink single crystals (yield: 0.96 g, 81%). FT–IR: 3630, 3405, 3209, 2286, 2069, 1612, 1535, 1446, 1400, 1181, 1114, 1031, 893, 857, 827, 758, 690, 615, 570, 490, 478, 401.

## Refinement   

The experimental details including the crystal data, data collection and refinement are summarized in Table 2 ▸. H atoms of water mol­ecules were located in difference-Fourier maps and refined with distance and angle restraints (SIMU, DELU and ISOR restraints in *SHELXL*). Bond lengths and angles for water mol­ecules are: O3—H31 = 0.806 (19), O3—H32 = 0.818 (18), O4—H41 = 0.827 (18), O5—H51 = 0.812 (10), O5—H52 = 0.820 (10) Å and H31—O3—H32 = 107 (4) and H51—O5—H52 = 107 (4)° The C-bound H atoms were positioned geometrically with C—H = 0.93 and 0.96 Å for aromatic and methyl H atoms, respectively, and constrained to ride on their parent atoms, with *U*_iso_(H) = *k* × *U*_eq_(C), where *k* = 1.5 for methyl H atoms and *k* = 1.2 for aromatic H atoms. The maximum and minimum electron densities were found 0.89 Å and 0.82 Å from Co1. The high residual electron density value of 2.178 e Å^−1^ may be due to the poor quality of the crystal.

## Supplementary Material

Crystal structure: contains datablock(s) I, global. DOI: 10.1107/S2056989017005564/pj2043sup1.cif

Structure factors: contains datablock(s) I. DOI: 10.1107/S2056989017005564/pj2043Isup2.hkl

CCDC reference: 1543701

Additional supporting information:  crystallographic information; 3D view; checkCIF report

## Figures and Tables

**Table 1:** Hydrogen-bond geometry (Å, °) *Cg*1 is the centroid of the C2–C7 ring.

*D*—H⋯*A*	*D*—H	H⋯*A*	*D*⋯*A*	*D*—H⋯*A*
O3—H31⋯O1^i^	0.80 (2)	1.90 (2)	2.697 (3)	170 (5)
O3—H32⋯O5	0.82 (3)	1.91 (3)	2.724 (5)	174 (3)
O4—H41⋯O2^ii^	0.83 (3)	1.82 (3)	2.622 (3)	164 (4)
O5—H52⋯O2^iii^	0.82 (3)	1.98 (4)	2.726 (4)	151 (6)
C10—H10*C*⋯O5^iv^	0.96	2.59	3.466 (7)	152
C6—H6⋯*Cg*1^v^	0.93	3.28	4.063 (4)	143
C9—H9*A*⋯*Cg*1^v^	0.96	3.40	3.961 (7)	120

Symmetry codes: (i) 
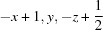
; (ii) 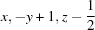; (iii) 
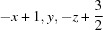
; (iv) 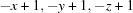; (v) 
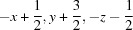
.

**Table 2:** Experimental details

Crystal data	
Chemical formula	[Co(C_10_H_11_O_2_)_2_(H_2_O)_3_]·2H_2_O
*M*_r_	475.39
Crystal system, space group	Monoclinic, *C*2/*c*
Temperature (K)	296
*a*, *b*, *c* (Å)	29.5261 (5), 10.1413 (2), 8.0906 (2)
β (°)	91.894 (4)
*V* (Å^3^)	2421.27 (9)
*Z*	4
Radiation type	Mo *K*α
μ (mm^−1^)	0.75
Crystal size (mm)	0.35 × 0.29 × 0.20

Data collection	
Diffractometer	Bruker SMART BREEZE CCD diffractometer
Absorption correction	Multi-scan *SADABS*; Bruker, 2012 ▸
*T*_min_, *T*_max_	0.779, 0.864
No. of measured, independent and observed [*I* > 2σ(*I*)] reflections	25445, 2957, 2448
*R*_int_	0.058
(sin θ/λ)_max_ (Å^−1^)	0.664

Refinement	
*R*[*F* ^2^ > 2σ(*F* ^2^)], *wR*(*F* ^2^), *S*	0.060, 0.166, 1.09
No. of reflections	2957
No. of parameters	161
No. of restraints	74
H-atom treatment	H atoms treated by a mixture of independent and constrained refinement
Δρ_max_, Δρ_min_ (e Å^−3^)	2.18, −0.52

Computer programs: *APEX2* and *SAINT* (Bruker, 2012 ▸), *SHELXS97* and *SHELXL97* (Sheldrick, 2008 ▸), *ORTEP-3 for Windows* and *WinGX* (Farrugia, 2012 ▸) and *PLATON* (Spek, 2009 ▸).

## supplementary crystallographic information

### Crystal data

**Table:**

[Co(C_10_H_11_O_2_)_2_(H_2_O)_3_]·2H_2_O	*F*(000) = 1004
*M**_r_* = 475.39	*D*_x_ = 1.304 Mg m^−^^3^
Monoclinic, *C*2/*c*	Mo *K*α radiation, λ = 0.71073 Å
Hall symbol: -C 2yc	Cell parameters from 9882 reflections
*a* = 29.5261 (5) Å	θ = 2.8–28.1°
*b* = 10.1413 (2) Å	µ = 0.75 mm^−^^1^
*c* = 8.0906 (2) Å	*T* = 296 K
β = 91.894 (4)°	Block, translucent light pink
*V* = 2421.27 (9) Å^3^	0.35 × 0.29 × 0.20 mm
*Z* = 4	

### Data collection

**Table:**

Bruker SMART BREEZE CCD diffractometer	2957 independent reflections
Radiation source: fine-focus sealed tube	2448 reflections with *I* > 2σ(*I*)
Graphite monochromator	*R*_int_ = 0.058
φ and ω scans	θ_max_ = 28.2°, θ_min_ = 1.4°
Absorption correction: multi-scan *SADABS*; Bruker, 2012	*h* = −38→39
*T*_min_ = 0.779, *T*_max_ = 0.864	*k* = −12→13
25445 measured reflections	*l* = −10→10

### Refinement

**Table:**

Refinement on *F*^2^	Primary atom site location: structure-invariant direct methods
Least-squares matrix: full	Secondary atom site location: difference Fourier map
*R*[*F*^2^ > 2σ(*F*^2^)] = 0.060	Hydrogen site location: inferred from neighbouring sites
*wR*(*F*^2^) = 0.166	H atoms treated by a mixture of independent and constrained refinement
*S* = 1.09	*w* = 1/[σ^2^(*F*_o_^2^) + (0.0725*P*)^2^ + 9.9721*P*] where *P* = (*F*_o_^2^ + 2*F*_c_^2^)/3
2957 reflections	(Δ/σ)_max_ < 0.001
161 parameters	Δρ_max_ = 2.18 e Å^−^^3^
74 restraints	Δρ_min_ = −0.52 e Å^−^^3^

### Special details

**Table:**

Geometry. All esds (except the esd in the dihedral angle between two l.s. planes) are estimated using the full covariance matrix. The cell esds are taken into account individually in the estimation of esds in distances, angles and torsion angles; correlations between esds in cell parameters are only used when they are defined by crystal symmetry. An approximate (isotropic) treatment of cell esds is used for estimating esds involving l.s. planes.
Refinement. Refinement of F^2^ against ALL reflections. The weighted R-factor wR and goodness of fit S are based on F^2^, conventional R-factors R are based on F, with F set to zero for negative F^2^. The threshold expression of F^2^ > 2sigma(F^2^) is used only for calculating R-factors(gt) etc. and is not relevant to the choice of reflections for refinement. R-factors based on F^2^ are statistically about twice as large as those based on F, and R- factors based on ALL data will be even larger.

### Fractional atomic coordinates and isotropic or equivalent isotropic displacement parameters (Å^2^)

**Table:**

	*x*	*y*	*z*	*U*_iso_*/*U*_eq_	
Co1	0.5000	0.5000	0.5000	0.02399 (18)	
O1	0.55057 (8)	0.6264 (2)	0.4251 (3)	0.0409 (6)	
O2	0.57111 (9)	0.7216 (3)	0.6626 (3)	0.0503 (7)	
O3	0.45248 (10)	0.6292 (3)	0.4084 (3)	0.0492 (7)	
H31	0.4484 (17)	0.625 (6)	0.310 (2)	0.086 (17)*	
H32	0.4289 (10)	0.656 (4)	0.446 (5)	0.057 (13)*	
O4	0.5000	0.4132 (3)	0.2500	0.0274 (6)	
H41	0.5249 (8)	0.378 (4)	0.237 (5)	0.048 (10)*	
O5	0.37711 (14)	0.7250 (4)	0.5521 (4)	0.0768 (10)	
H51	0.3607 (18)	0.785 (4)	0.522 (7)	0.103 (15)*	
H52	0.385 (2)	0.740 (6)	0.649 (3)	0.100 (15)*	
C1	0.57564 (11)	0.7012 (3)	0.5121 (4)	0.0348 (6)	
C2	0.61375 (11)	0.7694 (3)	0.4254 (4)	0.0366 (7)	
C3	0.65665 (13)	0.7129 (4)	0.4284 (5)	0.0509 (9)	
C4	0.69080 (15)	0.7756 (5)	0.3429 (6)	0.0625 (11)	
H4	0.7196	0.7386	0.3439	0.075*	
C5	0.68271 (16)	0.8917 (5)	0.2564 (5)	0.0647 (12)	
C6	0.64030 (15)	0.9461 (4)	0.2567 (5)	0.0567 (11)	
H6	0.6349	1.0245	0.1997	0.068*	
C7	0.60467 (13)	0.8874 (4)	0.3403 (4)	0.0450 (8)	
C8	0.55866 (16)	0.9483 (5)	0.3372 (7)	0.0689 (12)	
H8A	0.5409	0.9133	0.2458	0.103*	
H8B	0.5614	1.0421	0.3252	0.103*	
H8C	0.5441	0.9286	0.4387	0.103*	
C9	0.7209 (2)	0.9563 (8)	0.1634 (8)	0.109 (2)	
H9A	0.7152	0.9467	0.0465	0.164*	
H9B	0.7491	0.9145	0.1941	0.164*	
H9C	0.7225	1.0482	0.1911	0.164*	
C10	0.66644 (18)	0.5874 (5)	0.5233 (7)	0.0777 (15)	
H10A	0.6629	0.6028	0.6391	0.117*	
H10B	0.6970	0.5597	0.5049	0.117*	
H10C	0.6457	0.5198	0.4860	0.117*	

### Atomic displacement parameters (Å^2^)

**Table:**

	*U*^11^	*U*^22^	*U*^33^	*U*^12^	*U*^13^	*U*^23^
Co1	0.0380 (3)	0.0218 (3)	0.0124 (3)	−0.00099 (18)	0.00338 (18)	−0.00010 (16)
O1	0.0549 (14)	0.0470 (13)	0.0210 (9)	−0.0202 (10)	0.0055 (9)	−0.0019 (9)
O2	0.0670 (16)	0.0616 (16)	0.0227 (10)	−0.0243 (13)	0.0073 (10)	−0.0082 (10)
O3	0.0633 (16)	0.0626 (17)	0.0221 (11)	0.0257 (13)	0.0050 (10)	0.0023 (11)
O4	0.0375 (15)	0.0275 (13)	0.0175 (12)	0.000	0.0044 (10)	0.000
O5	0.087 (2)	0.094 (3)	0.0492 (18)	0.032 (2)	−0.0007 (16)	−0.0010 (18)
C1	0.0459 (16)	0.0352 (15)	0.0234 (13)	−0.0057 (12)	0.0017 (11)	0.0016 (11)
C2	0.0439 (16)	0.0405 (16)	0.0255 (13)	−0.0142 (13)	0.0024 (11)	−0.0034 (12)
C3	0.051 (2)	0.056 (2)	0.046 (2)	−0.0079 (17)	0.0059 (15)	0.0018 (17)
C4	0.048 (2)	0.084 (3)	0.056 (2)	−0.011 (2)	0.0104 (17)	0.005 (2)
C5	0.063 (3)	0.088 (3)	0.043 (2)	−0.033 (2)	0.0041 (18)	0.011 (2)
C6	0.072 (3)	0.057 (2)	0.0398 (19)	−0.027 (2)	−0.0063 (17)	0.0137 (17)
C7	0.055 (2)	0.0468 (19)	0.0326 (16)	−0.0138 (15)	−0.0030 (14)	0.0054 (14)
C8	0.071 (3)	0.060 (3)	0.075 (3)	0.002 (2)	−0.005 (2)	0.024 (2)
C9	0.081 (4)	0.163 (6)	0.083 (4)	−0.057 (4)	0.011 (3)	0.042 (4)
C10	0.070 (3)	0.068 (3)	0.095 (4)	0.015 (2)	0.015 (3)	0.025 (3)

### Geometric parameters (Å, º)

**Table:**

Co1—O1	2.074 (2)	C3—C10	1.509 (6)
Co1—O1^i^	2.074 (2)	C4—C5	1.387 (7)
Co1—O3	2.041 (2)	C4—H4	0.9300
Co1—O3^i^	2.041 (2)	C5—C9	1.524 (6)
Co1—O4	2.2060 (11)	C6—C5	1.368 (7)
Co1—O4^i^	2.2060 (11)	C6—H6	0.9300
O1—C1	1.259 (4)	C7—C6	1.402 (5)
O2—C1	1.246 (4)	C7—C8	1.492 (6)
O3—H31	0.806 (19)	C8—H8A	0.9600
O3—H32	0.818 (18)	C8—H8B	0.9600
O4—Co1^ii^	2.2060 (11)	C8—H8C	0.9600
O4—H41	0.827 (18)	C9—H9A	0.9600
O5—H51	0.812 (10)	C9—H9B	0.9600
O5—H52	0.820 (10)	C9—H9C	0.9600
C2—C1	1.513 (4)	C10—H10A	0.9600
C2—C3	1.390 (5)	C10—H10B	0.9600
C2—C7	1.401 (5)	C10—H10C	0.9600
C3—C4	1.395 (5)		
			
O1—Co1—O1^i^	180.00 (9)	C4—C3—C10	120.5 (4)
O1—Co1—O4	87.53 (7)	C3—C4—H4	119.3
O1^i^—Co1—O4	92.47 (7)	C5—C4—C3	121.5 (4)
O1—Co1—O4^i^	92.47 (7)	C5—C4—H4	119.3
O1^i^—Co1—O4^i^	87.53 (7)	C4—C5—C9	119.7 (5)
O3—Co1—O1	89.44 (11)	C6—C5—C4	118.9 (4)
O3^i^—Co1—O1	90.56 (11)	C6—C5—C9	121.4 (5)
O3—Co1—O1^i^	90.56 (11)	C5—C6—C7	122.1 (4)
O3^i^—Co1—O1^i^	89.44 (11)	C5—C6—H6	119.0
O3^i^—Co1—O3	180.00 (12)	C7—C6—H6	119.0
O3—Co1—O4	86.80 (8)	C2—C7—C6	117.7 (4)
O3^i^—Co1—O4	93.20 (8)	C2—C7—C8	121.4 (3)
O3—Co1—O4^i^	93.20 (8)	C6—C7—C8	120.9 (4)
O3^i^—Co1—O4^i^	86.80 (8)	C7—C8—H8A	109.5
O4^i^—Co1—O4	180.0	C7—C8—H8B	109.5
C1—O1—Co1	128.67 (19)	C7—C8—H8C	109.5
Co1—O3—H32	131 (3)	H8A—C8—H8B	109.5
Co1—O3—H31	114 (4)	H8A—C8—H8C	109.5
H32—O3—H31	107 (4)	H8B—C8—H8C	109.5
Co1—O4—Co1^ii^	132.95 (13)	C5—C9—H9A	109.5
Co1—O4—H41	108 (3)	C5—C9—H9B	109.5
Co1^ii^—O4—H41	92 (3)	C5—C9—H9C	109.5
H51—O5—H52	107 (4)	H9A—C9—H9B	109.5
O1—C1—C2	116.7 (3)	H9A—C9—H9C	109.5
O2—C1—O1	124.5 (3)	H9B—C9—H9C	109.5
O2—C1—C2	118.9 (3)	C3—C10—H10A	109.5
C3—C2—C1	119.7 (3)	C3—C10—H10B	109.5
C3—C2—C7	121.3 (3)	C3—C10—H10C	109.5
C7—C2—C1	119.0 (3)	H10A—C10—H10B	109.5
C2—C3—C4	118.5 (4)	H10A—C10—H10C	109.5
C2—C3—C10	121.1 (4)	H10B—C10—H10C	109.5
			
O3—Co1—O1—C1	105.4 (3)	C1—C2—C3—C10	−2.7 (6)
O3^i^—Co1—O1—C1	−74.6 (3)	C7—C2—C3—C4	−0.7 (6)
O4—Co1—O1—C1	−167.8 (3)	C7—C2—C3—C10	178.7 (4)
O4^i^—Co1—O1—C1	12.2 (3)	C1—C2—C7—C6	−178.0 (3)
O1—Co1—O4—Co1^ii^	−46.09 (7)	C1—C2—C7—C8	1.5 (5)
O1^i^—Co1—O4—Co1^ii^	133.91 (7)	C3—C2—C7—C6	0.6 (5)
O3—Co1—O4—Co1^ii^	43.49 (9)	C3—C2—C7—C8	−179.9 (4)
O3^i^—Co1—O4—Co1^ii^	−136.51 (9)	C2—C3—C4—C5	0.1 (7)
Co1—O1—C1—O2	−7.3 (5)	C10—C3—C4—C5	−179.3 (5)
Co1—O1—C1—C2	172.4 (2)	C3—C4—C5—C6	0.6 (7)
C3—C2—C1—O1	−94.3 (4)	C3—C4—C5—C9	−179.5 (5)
C3—C2—C1—O2	85.4 (4)	C7—C6—C5—C4	−0.7 (7)
C7—C2—C1—O1	84.4 (4)	C7—C6—C5—C9	179.4 (5)
C7—C2—C1—O2	−95.9 (4)	C2—C7—C6—C5	0.1 (6)
C1—C2—C3—C4	177.9 (3)	C8—C7—C6—C5	−179.4 (4)

Symmetry codes: (i) −*x*+1, −*y*+1, −*z*+1; (ii) −*x*+1, *y*, −*z*+1/2.

### Hydrogen-bond geometry (Å, º)

Cg1 is the centroid of the C2–C7 ring.

**Table:**

*D*—H···*A*	*D*—H	H···*A*	*D*···*A*	*D*—H···*A*
O3—H31···O1^ii^	0.80 (2)	1.90 (2)	2.697 (3)	170 (5)
O3—H32···O5	0.82 (3)	1.91 (3)	2.724 (5)	174 (3)
O4—H41···O2^iii^	0.83 (3)	1.82 (3)	2.622 (3)	164 (4)
O5—H52···O2^iv^	0.82 (3)	1.98 (4)	2.726 (4)	151 (6)
C10—H10*C*···O5^i^	0.96	2.59	3.466 (7)	152
C6—H6···*Cg*1^v^	0.93	3.28	4.063 (4)	143
C9—H9*A*···*Cg*1^v^	0.96	3.40	3.961 (7)	120

Symmetry codes: (i) −*x*+1, −*y*+1, −*z*+1; (ii) −*x*+1, *y*, −*z*+1/2; (iii) *x*, −*y*+1, *z*−1/2; (iv) −*x*+1, *y*, −*z*+3/2; (v) −*x*+1/2, *y*+3/2, −*z*−1/2.
